# The Utilization of Natural Language Processing for Analyzing Social Media Data in Nursing Research: A Scoping Review

**DOI:** 10.1155/jonm/2857497

**Published:** 2024-12-30

**Authors:** Zhenrong Wang, Yulin Ma, Yuanyuan Song, Yao Huang, Guopeng Liang, Xi Zhong

**Affiliations:** ^1^Department of Pulmonary and Critical Care Medicine, West China Hospital, Sichuan University, Chengdu 610041, Sichuan, China; ^2^State Key Laboratory of Respiratory Health and Multimorbidity, West China Hospital, Sichuan University, Chengdu 610041, Sichuan, China; ^3^School of Computer and Artificial Intelligence, Southwest Jiaotong University, Chengdu 611730, Sichuan, China; ^4^Department of Critical Care Medicine, West China Hospital, Sichuan University/West China School of Nursing, Sichuan University, Chengdu 610041, Sichuan, China; ^5^State Key Laboratory of Oral Diseases & National Clinical Research Center for Oral Diseases & Department of Cariology and Endodontics, West China Hospital of Stomatology, Sichuan University, Chengdu 610041, Sichuan, China; ^6^Department of Respiratory Care, West China Hospital, Sichuan University, Chengdu 610041, Sichuan, China; ^7^Department of Critical Care Medicine, West China Hospital, Sichuan University, Chengdu 610041, Sichuan, China

**Keywords:** latent Dirichlet allocation, natural language processing, nursing, sentiment analysis, social media, topic modeling

## Abstract

**Aim:** This scoping review aimed to identify and synthesize the evidence in existing nursing studies that used natural language processing to analyze social media data, and the relevant procedures, techniques, tools, and ethical issues.

**Background:** Social media has widely integrated into both everyday life and the nursing profession, resulting in the accumulation of extensive nursing-related social media data. The analysis of such data facilitates the generation of evidence thereby aiding in the formation of better policies. Natural language processing has emerged as a promising methodology for analyzing social media data in the field of nursing. However, the extent of natural language processing applications in analyzing nursing-related social media data remains unknown.

**Evaluation:** A scoping review was conducted. PubMed, CINAHL, Web of Science and IEEE Xplore were searched. Studies were screened based on inclusion criteria. Relevant data were extracted and summarized using a descriptive approach.

**Key Issues:** In total, 38 studies were included for the final analysis. Topic modeling and sentiment analysis were the most frequently employed natural language processing techniques. The most used topic modeling algorithm was latent Dirichlet allocation. The dictionary-based approach was the most utilized sentiment analysis approach, and the National Research Council Sentiment and Emotion Lexicons was the most used sentiment dictionary. Natural language processing tools such as Python (*NLTK, Jieba, spaCy,* and *KoNLP* library) and R (*LDAvis*, *Jaccard, ldatuning,* and *SentiWordNet* packages) were documented. A significant proportion of the included studies did not obtain ethical approval and did not conduct data anonymization on social media users' information.

**Conclusion:** This scoping review summarized the extent of natural language processing techniques adoption in nursing and relevant procedures and tools, offering valuable resources for researchers who are interested in discovering knowledge from social media data. The study also highlighted that the application of natural language processing for analyzing nursing-related social media data is still emerging, indicating opportunities for future methodological improvements.

**Implications for Nursing Management:** There is a need for a standardized management framework for conducting and reporting studies using natural language processing techniques in the analysis of nursing-related social media data. The findings could inform the development of regulatory policies by nursing authorities.

## 1. Introduction

Social media is defined as computer-mediated technologies that facilitate the creation and sharing of user-generated content via virtual communities and networks [1]. Popular social media platforms include Facebook, YouTube, WhatsApp, Instagram, WeChat, Twitter, and TikTok. The use of social media has grown exponentially during the past decades and it has become inseparable from people's everyday life. In April 2017, Facebook boasted 2.4 billion monthly active users [2]. Within the vast social media user base are healthcare professionals [3, 4], clients, family caregivers, and other healthcare stakeholders. Healthcare professionals are increasingly recognizing the potential of social media and applying it in various healthcare settings [5, 6]. In the field of nursing, social media is utilized for information acquisition and dissemination, nursing intervention implementation, patient education, and nursing research purposes.

The widespread use of social media in the field of nursing has contributed to the generation of extensive nursing-related social media data. The abundance of social media data offers new perspectives for nursing, providing valuable information on public perceptions and voices of clients and their families [7, 8]. Nursing researchers have leveraged social media data to detect client and family experiences and care needs, investigate public perceptions of health issues, explore the reputation of the nursing profession, and delve into the narratives of nursing professionals and student nurses [7–11]. These nursing-related social media data come in various formats, including texts, images, videos, emojis, and hyperlinks. It is a representative example of unstructured big data, characterized by its large quantity, diverse formats, and constantly evolving nature. The substantial volume and diverse formats of social media data have the potential to generate new evidence and support informed decision-making, knowledge discovery, and process optimization in nursing practice. However, the high volume, high velocity, and high variety characteristics of social media data pose challenges to conventional statistical analysis methods. To address these challenges and enhance data processing efficiency, nursing researchers are increasingly turning to advanced data processing and analytic techniques such as natural language processing (NLP) techniques.

NLP is “the subfield of computer science and artificial intelligence that is concerned with using computational techniques to learn, understand, and produce human language content” [12]. An NLP pipeline typically consists of three main components: data preprocessing, feature extraction, and modeling [13]. Data preprocessing includes tasks such as tokenization, stemming, lemmatization, and stop word removal, which converts raw input text into cleansed tokens, thereby reducing the dimensionality of the text dataset. Feature extraction plays a crucial role in NLP by supporting downstream tasks through the application of various algorithms, such as bag-of-words, term frequency–inverse document frequency (TF-IDF), and word embedding. The extracted features were referred to as contextual and noncontextual embeddings that may encompass features such as lexical meanings, semantic features, and syntactic features. Subsequently, models for specific NLP tasks are constructed using the extracted features to produce the desired outputs. NLP enables the extraction of valuable knowledge from unstructured healthcare data, thereby facilitating informed decision-making and ultimately leading to improved patient outcomes.

Methodologies for analyzing social media data in the healthcare discipline have been documented. Tsao et al. [14] examined studies utilizing social media data to explore public attitudes, identify infodemics, assess mental health issues, and detect or predict COVID-19 cases, and mentioned the application of NLP techniques; yet there was a lack of detailed descriptions of the employed NLP techniques. Bour et al. [15] investigated studies that utilized social media for health purposes and found that, while a subset of the results presented the application of NLP techniques in certain included studies, the detailed methodology approach warrants further exploration. A scoping review focused on the use of blog data for health research, but the emphasis was on studies employing qualitative methods and the use of blogs as recruitment tools [16]. Fu et al. [17] conducted a review of the methodologies utilized in analyzing healthcare-related social media content, revealing that qualitative content analysis emerged as the predominant methodology despite the presence of NLP techniques. Recently, a rapid review was published on the current and potential use of large language models, a product of NLP, in nursing, highlighting new directions for the nursing discipline [18]. To summarize, the use of NLP techniques for analyzing nursing-related social media has been documented, and there are review studies that focus on healthcare social media analytics. However, these studies tend to have comprehensive scopes, and the documented methodologies varied, encompassing qualitative, quantitative, and traditional statistical approaches as well as advanced computational methodologies such as NLP. Therefore, the extent to which NLP techniques are applied in social media analysis within the nursing field, the specific NLP techniques employed, their implementation procedures, associated tools, and related ethical considerations, warrant further exploration.

This scoping review is intended to synthesize the presently available state-of-the-art research that focuses on the use of NLP techniques for processing social media data in the nursing discipline. This scoping review will (1) identify and describe the studies that employed NLP for analyzing social media data in the nursing discipline, (2) explore the characteristics of analyzed data, (3) provide a detailed summary of the NLP approaches, techniques, and tools used in included studies, (4) and discuss the ethical issues in adopting NLP for analyzing nursing-related social media data.

## 2. Methods

This scoping review encompassed studies focusing on the application of NLP techniques for analyzing and processing social media data within the realm of nursing research. There is a prolific body of literature using social media–generated data in healthcare research, and the definition of nursing research could be broad, as any healthcare-related topic is potentially related to nursing. For the purposes of this review, nursing research was narrowed down to research conducted by researchers with nursing backgrounds; research published in nursing journals irrespective of the background of researchers; or research explicitly emphasizing its relevance to clinical nursing practice, nursing education, or nursing research. The background of the authors was determined through the affiliation information presented in the articles and author profiles on ResearchGate (https://www.researchgate.net). This scoping review conducted aligns with the methodological framework proposed by Arksey and O'Malley [19] and is refined by Levac, Colquhoun, and O'Brien [20]. The methodological framework involves five stages: (1) identifying and linking research questions and purposes; (2) identifying relevant studies; (3) using a team approach to selecting studies; (4) extracting and charting data; (5) reporting results that incorporate a numerical summary and qualitative thematic analysis; and stating the implications of study results to policy, practice, and research [20].

This scoping review is reported following the Preferred Reporting Items for Systematic Reviews and Meta-Analyses extension for Scoping Reviews (PRISMA-ScR) checklist [21]. In adherence to the PRISMA-ScR checklist and given that only publicly accessible journal articles are used to derive evidence, institutional ethical approval was not required for this study. A protocol of this review is available at the OSF website (https://osf.io/kjstq), registration doi: https://doi.org/10.930429/kjstq.

### 2.1. Identifying the Research Questions

The objective of this scoping review was to identify and elucidate the current literature concerning the application of NLP techniques for analyzing social media data within the nursing domain. The research aimed to address the following research questions:

1. To what extent are NLP techniques employed for the analysis of social media data in the field of nursing?
2. What are the key characteristics of social media data that have been processed using NLP techniques in nursing research?
3. What specific NLP algorithms and computational tools have been utilized for processing social media data in nursing studies?
4. What ethical considerations are associated with the application of NLP techniques for analyzing social media data in the context of nursing research?

### 2.2. Identifying Relevant Studies

To develop a robust search strategy, an initial search was carried out in PubMed to identify pertinent terms. The final search strategy was formed and refined in collaboration with a health science librarian. The search was conducted across multiple databases including PubMed (MEDLINE), Cumulative Index to Nursing and Allied Health Literature (CINAHL), Web of Science, Embase, Institute of Electrical and Electronics Engineers Digital Library (IEEE Xplore), and ScienceDirect, spanning from the inception of the databases to November 10, 2023. Our search strategy consists of medical subject headings (MeSH) and a series of free-text terms related to “nursing,” “social media,” and “NLP.” Initially executed in PubMed (Table 1), the search strategy was subsequently tailored for compatibility with other databases. To facilitate the review process and enhance result generalizability, only peer-reviewed articles published in English were included. To avoid missing papers, the reference lists of included studies were scanned to identify potentially related literature. An update search was conducted on April 2024 using the same search strategy.

### 2.3. Eligibility Criteria

The inclusion criteria of the studies were as follows: (1) NLP techniques were utilized to analyze social media data as explicitly stated in the Methods section, (2) studies have explicit relevance to nursing practice, (3) primary peer-reviewed studies, and (4) studies published in the English language.

Exclusion criteria were as follows: (1) the analyzed social media data were generated by researcher-initiated social media pages for research purposes, (2) NLP techniques were not mentioned in the method, (3) the analyzed dataset consists of data from sources other than social media platforms, and (4) conference papers.

### 2.4. Data Charting Process

Title and abstract of retrieved articles were exported to EndNote X9, where duplicates were removed. During the initial screening phase, the title and abstract of identified articles were screened independently by two reviewers and categorized as “include,” “exclude,” and “potentially include.” A third reviewer was introduced to resolve any discrepancies. Studies categorized as “potentially included” were further evaluated by all three reviewers to make a final decision. The authors developed a pilot data extraction template to systematically extract relevant information from the full text of the included studies. Two authors independently extracted data from a sample of 10 articles using the template and then met to discuss discrepancies and make necessary modifications. The refined data extraction form was then used for data charting. The extracted information from the included articles encompassed the following aspects:

1. Publication details: year of publication, journal name, and country of origin.
2. Author information: number of authors with nursing expertise and expertise of the first or corresponding author.
3. Data acquisition: social media platform used, sample size, data collection period, type of data analyzed, language of collected data, geological scope of collected data, inclusion and exclusion criteria, and methods for retrieving social media data [22] (keyword based, user based, hashtag based, and column-based).
4. Data analysis: software used for data analysis, data analysis techniques, and data analytic procedures.
5. Study topic: keywords, aim of research, specific disease topic under investigation, and key findings.
6. Ethical aspects: ethical approval and data anonymization.

Two reviewers independently extracted data from each included article. The research topic was first recorded in free texts according to the main concept of the study. Then, three authors derived categories from the recorded topics, and finally, the research topics of the included studies were categorized into the derived topics.

### 2.5. Collating, Summarizing, and Reporting the Results

The data and information were extracted to an Excel file and analyzed using descriptive statistics. Qualitative data were synthesized through content analysis. Scoping review synthesizes an extensive number of studies with various study designs, rather than merely randomized controlled trials. To align with the methodology of the scoping review, no formal quality assessment of the included studies was conducted [19].

## 3. Results

**Figure 1:**
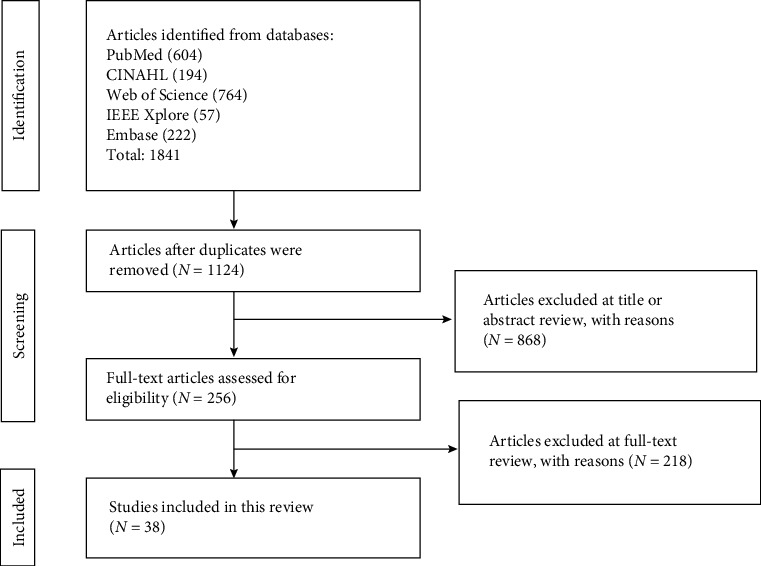
PRISMA flowchart.

As shown in the PRISMA flowchart diagram (Figure 1), a total of 1841 articles were identified from databases. Altogether, 717 duplicates were removed and 868 were excluded during the title and abstract screening process. Finally, 38 studies that met the inclusion criterion were included in this review.

### 3.1. Characteristics of Included Studies

The included studies, published between 2019 and 2023, involved researchers from various countries: U.S.A. (*n* = 22), Republic of Korea (*n* = 6), China (*n* = 5), Turkey (*n* = 3), Australia (*n* = 1), and Israel (*n* = 1). As for the background of researchers, 32% (*n* = 12) of the studies were published by authors with nursing background exclusively, but the majority of the studies (*n* = 26, 68%) were conducted by multidisciplinary teams comprising individuals with nursing backgrounds and expertise in medicine, computer science, information science, psychiatry, or social work. The first author or corresponding authors with nursing backgrounds accounted for 74% (*n* = 28) of the included studies. In some studies (*n* = 11, 29%), qualitative methods such as manual coding, content analysis, thematic analysis, and manual annotation were utilized to facilitate the NLP process.

### 3.2. Research Topics of Included Studies

The research topics of the included studies were mainly focused on the following four aspects: nursing-related health/disease topics (*n* = 22), experiences of nurses (*n* = 7), sentiments regarding the nursing profession (*n* = 5), and experiences of patients or caregivers (*n* = 4). Nursing-related health/disease topics encompassed various issues, such as cancer, dementia, Alzheimer's disease, COVID-19 and its vaccination, Ebola, autism spectrum disorders, substance use, suicide, and other chronic illnesses. Among the studies that focused on the nursing profession, 8 out of 10 studies were centered on the COVID-19 pandemic period. In this review, 13 studies analyzed social media posts collected during the COVID-19 pandemic, exploring themes like sentiments toward COVID-19 vaccination, experiences of COVID-19-positive individuals, and the experiences and mental aspects of nursing professionals during the pandemic.

### 3.3. Characteristics of the Analyzed Social Media Dataset

Most of the analyzed social media content was extracted from Twitter (55%, *n* = 21). The sample size of the included studies varied significantly, ranging from 1569 to 137 million pieces of social media posts. Notably, in approximately 32% (*n* = 12) of the studies, more than 100,000 pieces of social media posts were examined. In most of the included studies, the sample dataset comprised over 10,000 social media posts. English texts were the most commonly analyzed data form across all the included studies. It is worth noting that the datasets were predominantly extracted in large quantities without preset selection criteria, except for 9 studies where the sample datasets were obtained based on researcher-defined inclusion and exclusion criteria. The most used social media data retrieving method (*n* = 21, 55%) was keywords-based searching. Detailed information related to the characteristics of the analyzed social media dataset is shown in Table 2.

### 3.4. NLP Methodologies in Included Studies: Data Collection Methods, Text Preprocessing, and NLP Approaches

Detailed descriptions of data collection and extraction methods/processes were available in around half (*n* = 17, 48.6%) of the included studies. Data collection tool was reported in 76% (*n* = 26) of the included studies. Python was the most commonly used data collection tool (*n* = 13), followed by R (*n* = 4), and NCapture (*n* = 4). Specifics of data collection tools are shown in Table 3.

Approximately 66% (*N* = 25) of the included studies indicated that text preprocessing was performed, with 60% (14/24) of them providing the details of text preprocessing. Frequently mentioned text preprocessing tools included Python libraries such as *Natural Language Toolkit (NLTK)* [23–25], *Jieba* [26, 27]*, spaCy* [28], and R package *tm* [29, 30]. In some studies, it was noted that data preprocessing was carried out manually without the aid of computational techniques, as the specific computational tools were not specified despite mentioning text preprocessing.

Of the included studies, topic modeling and sentiment analysis were the most frequently used NLP approaches to explore nursing-related social media content. More than half of the studies (58%, *n* = 22) utilized topic modeling NLP approach and 55% (*n* = 21) studies utilized sentiment analysis. Notably, 37% (*n* = 14) of the included studies employed topic modeling and sentiment analysis NLP approaches in combination. Furthermore, eight studies utilized topic modeling either alone or in conjunction with conventional analytic methods such as statistical analysis and qualitative content analysis. In addition, seven studies used sentiment analysis alone or in combination with descriptive statistics and qualitative content analysis. Details about NLP techniques, data processing tools, and data analysis process of the included studies are summarized in Table 3.

Topic modeling is a technique that encompasses a group of algorithms that reveal, discover, and annotate thematic structure in text. Topic models are categorized into algebraic, fuzzy, Bayesian probabilistic, and neural topic models. Popular topic modeling algorithms included latent Dirichlet allocation (LDA), non-negative matrix factorization (NMF), Top2Vec, and BERTopic [31]. Topic modeling was used to discover the hidden themes from social media text in 21/38 of the included studies [23, 26, 28, 30, 32–40]. Among the 22 studies, the LDA algorithm that belongs to the Bayesian probabilistic topic modeling approach was the most frequently used algorithm for topic modeling and was applied in 15 studies. For the rest two studies that conducted topic modeling, bag-of-words [41] and NMF [28] that pertain to an algebraic topic modeling approach, hierarchical density–based spatial clustering of applications with noise (HDBSCAN) were used. The commonly used topic modeling toolkit is Python genism library [23, 24, 38], Mallet [24, 33], R package *topicmodels* [29, 30, 42] and *ldatuning* [29, 30, 42]. In the reported studies, results of topic modeling were presented with word cloud [30, 33, 43], heatmap [28], LDAvis [34, 42], and qualitative content analysis [36, 37, 39, 44].

Sentiment analysis is an NLP technique that determines whether a piece of content is positive, negative, or neutral. Sentiment analysis was mainly achieved through three approaches: lexicon-based approach, machine learning approach, and hybrid approach [45]. For the lexicon-based approach, there are the dictionary-based approach and corpus-based approach. The machine learning approach included decision tree classifiers, linear classifiers, rule-based classifiers, probabilistic classifiers, and K-nearest neighbor. Of the included studies, 21 studies applied the sentiment analysis technique [23, 25, 26, 29, 30, 32, 34, 37–40, 43, 46–52], with 17 of them documenting the exact approach used. The most frequently used sentiment analysis approach was the dictionary-based approach, utilized in 15 studies. The National Research Council (NRC) Sentiment and Emotion Lexicons was the most widely adopted dictionaries, employed in 6 studies [29, 30, 32, 34, 50, 51]. Other documented sentiment dictionaries were LIWC [32, 38], the Chinese DLUT-Emotion Ontology, the Chinese Emotion Valence Dictionary [26], SentiWordNet [49], AFINN [51, 52], and BING [51, 52]. In 3 studies [26, 51, 52], multiple sentiment dictionaries were utilized. In addition to the lexicon-based approach, the machine learning approach was used for sentiment analysis, including RNN [23, 40], Bi-LSTM [27], and Naive Bayes [25]. Some studies detected the utilization of more advanced transfer learning techniques such as BERT, which are faster and less costly to train [47, 48]. VADER was also mentioned in this context [23, 32, 53].

### 3.5. Ethical Issues in Reported Research

The disparity in obtaining ethical approval among the studies was observed. In this review, ethical considerations were mentioned in 89% (*n* = 34) of the studies. Among them, ethical approval was obtained in 37% (*n* = 14) of the studies, exempted in three studies, and not obtained in 45% (*n* = 17) of the studies. Data anonymization that eliminates social media user's identifiable information was conducted in a relatively limited number (*n* = 12, 32%) of studies utilizing NLP for processing collected social media text.

## 4. Discussion

Nursing researchers are currently adopting NLP techniques to explore social media content concerning nursing-related health topics, experience of nurses, sentiments about nursing profession, and the experiences of patients or caregivers. This scoping review highlighted the utilization of NLP techniques such as topic modeling and sentiment analysis to gain insights from social media content related to nursing. Among these techniques, the LDA algorithm was the most commonly used topic modeling algorithm. In addition, sentiment analysis approaches, including dictionary-based and machine learning methods, were frequently used to determine the tone of social media content regarding nursing and its associated themes. Analyzing social media data in the nursing domain using NLP techniques presents challenges, since it requires an interdisciplinary methodological framework that integrates advanced computational techniques, conventional quantitative and qualitative methodologies, and a profound understanding of nursing domain knowledge.

This scoping review showed that Twitter was the most prevalent data source of the included studies. Twitter, a widely used microblogging platform, enables users to share a 280-character short message, along with multimedia content such as photos and videos. With over 500 million tweets including health-related tweets being posted daily [54], Twitter has emerged as a valuable data source for nursing researchers and healthcare professionals seeking real-world insights. Previous studies have demonstrated the frequent use of Twitter to collect and analyze healthcare-related social media data. In a review by Tsao focusing on social media usage during the COVID-19 pandemic, Twitter was the predominant social media platform [14]. Reviews on social media analytics have consistently highlighted Twitter as the preferred platform for data collection due to its easy data accessibility [1, 55]. Twitter initiated the Twitter Data Grants program that encourages researchers to access historical data and public tweets, while other major platforms such as Facebook and Google are increasingly restricting data exportation [56]. Researchers benefit from Twitter's user-friendly application programming interfaces (APIs) for data retrieval, aligning with our finding that Twitter was the most commonly utilized data source in nursing studies employing NLP for social media analysis.

English was the most frequently analyzed text. Prior to the NLP process, various text preprocessing techniques were performed, including tokenization, word segmentation, part-of-speech tagging, and parsing. When it comes to tokenization, a process that splits sentences or documents into tokens (words or phrases), it is straightforward to split words in English since they are separated by spaces, yet more challenging for languages such as Chinese, Japanese, Korean, and others that lack explicit word boundary markers [57]. Consequently, tokenizing these languages requires advanced algorithms and models that can accurately identify word boundaries within the text.

It is noticeable that a good proportion of the research that adopted NLP techniques to process nursing-related social media data was concentrated on the COVID-19 topic. There was a surge in social media use during the COVID-19 pandemic, since the rapid dissemination of crisis-related news and the shift from face-to-face interaction to online conversations and discussions caused by quarantine. Notably, numerous research studies have highlighted a substantial increase in social media analytics studies during the onset of the COVID-19 period. This trend is exemplified by research projects such as the analysis of Twitter data to track public sentiment toward COVID-19 vaccination efforts, the examination of Facebook posts to understand the impact of the pandemic on mental health discussions in nursing communities, and the use of Instagram data to assess the dissemination of misinformation related to COVID-19 among healthcare professionals.

LDA is a generative probabilistic model that assumes each document as a random mixture of latent topics, each characterized by a distribution over words [58]. Initially introduced by Blei, Ng, and Jordan [59], LDA has become one of the most popular topic modeling methods. Consistent with Fu et al. [17] study where LDA was the primary computer-aided method for analyzing healthcare-related social media content, the LDA algorithm remains the predominant topic modeling approach in this review. Through topic modeling, nursing researchers were able to extract first-hand information from social media content shared by patients and their families in a cost-effective manner and summarize it into knowledge that has the potential to inform clinical nursing practice and policymaking. For example, by applying the LDA algorithm to analyze posts from an online forum, researchers found that the most frequently expressed symptoms experienced among ovarian cancer patients were pain, nausea, anxiety, fatigue, and skin rash [35], providing insights for the prioritization of patient care needs.

Despite the widespread application of the LDA algorithm in social media analytics, previous studies have suggested that it was more suitable for processing long text, given its reliance on word co-occurrence calculations. In short texts such as social media posts, however, only very limited word co-occurrence information is available. To tackle this issue, Qiang et al. [60] summarized current research and presented three methodology approaches based on Dirichlet multinomial mixture, global word co-occurrences, and self-aggregation and compared their performance versus the long text topic modeling algorithm. Moreover, they initiated an open-source Java library named Short Text Topic Modeling (STTM) (https://github.com/qiang2100/STTM) with good extendibility that allows future work to incorporate topics easily.

The dictionary-based approach was the most frequently used sentiment analysis approach identified in this study, and NRC Sentiment and Emotion Lexicons was the most popular dictionary. NRC Sentiment and Emotion Lexicons is a list of words (in various languages) that are associated with eight basic emotions (anger, fear, anticipation, trust, surprise, sadness, joy, and disgust) and two sentiments (negative and positive) [61]. The task was accomplished through mapping words in the dataset to the NRC Sentiment and Emotion Lexicons, checking if the word exists in the lexicon and determining the sentiment or emotion category it belongs to. The sentiment scores were calculated by summing up the scores of individual words. NRC Sentiment and Emotion Lexicons was usually used in conjunction with third-party software packages such as Python package *Emotion Dynamic* and *NLTK* [61]. In the study of Xavier and Lambert [51], the emotions and sentiment trends of self-identified nurses on Twitter during the COVID-19 pandemic were documented through a sentiment analysis approach that used the NRC lexicon, Bing lexicon, and AFINN lexicon. Several included studies revealed patients or caregivers' sentiments toward a specific disease, which provide insights to refine related nursing interventions for the targeted population [26, 39]. The dictionary-based approach is advantageous in that no pretrained data are needed, yet it is highly domain-specific, meaning that words relevant to one domain may not be applicable in another domain [62]. This brought researchers to the adoption of a machine learning approach that involves classification or regression tasks similar to where a model is trained using labeled data, requiring intensive computing. In this study, machine learning approaches such as RNN and its variant Bi-LSTM, which have been extensively employed in sentiment analysis and related NLP tasks, were also identified [23, 27, 45, 63].

In this review, a concerning number of studies proceeded without formal ethical approval or did not mention it. It is also worrisome that data anonymization was only mentioned in a relatively small proportion of the included studies. A similar situation was observed in studies focusing on the ethical considerations and methodological uses of social media data from platforms such as Facebook, Twitter, and YouTube in public health research [64]. It was revealed that in studies utilizing Facebook data for public health research, less than half of the studies gained ethical approval, and informed consent from users is scarce. Currently, there is still controversy over the ethical approval of conducting research using social media data, since it is generally argued that the data that can be collected is public, and in some cases freely available [65, 66]. While social media posts are publicly available, it is important to recognize that the mere accessibility of this information does not automatically imply ethical appropriateness and consent in its collection, analysis, and dissemination. This is particularly true in the nursing discipline, where research often focuses on sensitive health and personal information. Our findings suggested that there is a need to develop ethical guidelines to navigate the accountable use of nursing-related social media data, thereby ensuring the proper obtaining of informed consent, the protection of individuals' privacy, and promoting the ethical use of social media data in nursing practice and research.

This review highlighted the imminent need for a commonly accepted framework that can guide the conduction and reporting of studies utilizing NLP for the analysis of nursing or healthcare-related social media data. The reported methodological information of the included studies varied, with some studies offering comprehensive details while others lacked the information on data collection and analysis tools utilized. Stieglitz et al. [2] presented a social media analytics framework (SMAF) that describes a process consisting of three steps including tracking, preparation, and analysis. Moreover, He et al. [22] introduced a standardized protocol named Protocol of Analysis of Sentiment in Health (PATH) for computational sentiment analysis research using health-related social media data. PATH consists of three dimensions, including platform selection, data curation, and sentiment analysis method. A universally accepted reporting guideline plays a crucial role in ensuring that research studies are conducted in a consistent and reproducible manner, thereby maintaining the integrity of research. In addition, such a guideline would facilitate the comparison of findings across studies, promote transparency, and strengthen the ethical foundation of research practices.

This study has certain limitations, primarily stemming from the study design, which involved the use of a scoping review. For example, the review did not include a quality assessment of the included studies. Since the adoption of NLP for analyzing social media data in nursing is still in its emerging phase, quality assessment was omitted to ensure a more comprehensive understanding of this subject. Furthermore, the scope of this review was limited to articles published in English-language peer-reviewed journals, and conference papers were excluded. This limitation could potentially result in the omission of pertinent articles published in other languages. Hence, the NLP techniques employed to process languages other than English need further exploration. This gap presents an opportunity for nursing researchers to evaluate and compare the NLP techniques utilized for managing text across different languages. Apart from NLP techniques, qualitative methods were used extensively for analyzing nursing-related social media data. Future research should be conducted to explore the use of qualitative methods for analyzing social media data and to compare the effectiveness and outcomes between qualitative methods with NLP approaches.

## 5. Implications for Nursing Management

Several key implications emerged from the findings of this scoping review. The variations in the level of methodological detail reported in the included studies point out the need for a standardized framework or guideline for conducting and reporting studies utilizing NLP for the analysis of nursing-related social media data. Second, this study sheds light on the current landscape of ethical approval concerning the analysis of social media data in the nursing discipline. These findings could inform the development of regulatory policies by nursing governing bodies. Third, more educational opportunities should be provided for nurses to gain knowledge in NLP, so the profession has the necessary skills and knowledge to conduct and participate in relevant studies.

## 6. Conclusion

Social media data have increasingly been analyzed to facilitate knowledge discovery and policymaking in nursing, yet the landscape of NLP adoption in the analysis of nursing-related social media data remains unexplored. This review examines the characteristics of studies that employed NLP techniques, detailing the specifics of the adopted NLP approaches, algorithms, computational tools, and ethical issues involved. The dominant NLP approach used for processing nursing-related social media data includes topic modeling and sentiment analysis, with LDA being the most frequently used topic modeling algorithm. Popular data processing tools identified in the review include Python (*NLTK, Jieba, spaCy, MALLET,* and *KoNLP*) and R (*LDAvis*, *Jaccard, ldatuning,* and *SentiWordNet*). This scoping review offers an insightful understanding of the current landscape of NLP techniques utilization in analyzing nursing-related social media data. The methodological aspects summarized from the included studies, such as the data retrieval methods, text preprocessing techniques, and NLP approaches and their procedures and tools, serve as valuable resources for nursing researchers interested in social media analytics. In addition, the review highlights that the application of NLP techniques in nursing social media analysis is still in its nascent stages, suggesting ample opportunities for future methodological advancements and refinements.

## Figures and Tables

**Table 1:** Steps and detailed search terms used in PubMed.

Steps	Search terms
1	Subject area I: Nursing or nurse “Nursing” [MeSH terms] OR “nursing” [title/Abstract] OR “nurses” [MeSH terms] OR “nurses” [title/Abstract] OR “students, nursing” [MeSH terms] OR “nursing students” [title/Abstract]
2	Subject area II: NLP techniques “Data mining” [MeSH terms] OR “sentiment analysis” [MeSH terms] OR “natural language processing” [MeSH terms] OR “NLP” [title/Abstract] OR “text mining” [title/Abstract] OR “content analysis” [title/Abstract] OR “text analysis” [title/Abstract] OR “topic modeling” [title/Abstract] OR “social media analytics” [title/Abstract] OR “big data analytics” [title/Abstract] OR “social media big data” [title/Abstract] OR “machine learning” [MeSH terms] OR “opinion mining” [Title/Abstract] OR “machine learning algorithms” [Title/Abstract] OR “latent Dirichlet allocation”[Title/Abstract] OR “LDA” [title/Abstract] OR “deep learning” [MeSH terms] OR “deep learning” [title/Abstract] OR “clustering algorithm”[Title/Abstract] OR “active learning”[Title/Abstract] OR “big data” [MeSH terms] OR “big data” [title/Abstract]
3	Subject area III: Social media “Social media” [MeSH terms] OR “social media” [title/Abstract] OR “blogging” [MeSH terms] OR “blogging” [title/Abstract] OR “social networking sites” [title/Abstract] OR “social networks” [title/Abstract] OR “Web2.0” [title/Abstract] OR “twitter” [title/Abstract] OR “facebook” [title/Abstract] OR “Sina Weibo” [title/Abstract] OR “WeChat” [title/Abstract] OR “YouTube” [title/Abstract] OR “TikTok” [title/Abstract] OR “Instagram” [title/Abstract] OR “WhatsApp” [title/Abstract] OR “Snapchat” [title/Abstract] OR “Reddit” [title/Abstract] OR “online post” [title/Abstract] OR “online discussion” [title/Abstract] OR “online community” [title/Abstract] OR “posting” [title/Abstract] OR “blog” [title/Abstract] OR “microblog” [title/Abstract] OR “tweet” [title/Abstract] OR “retweet” [title/Abstract] OR “webcast” [title/Abstract] OR “user-generated content” [title/Abstract])

**Table 2:** Characteristics of analyzed social media datasets of included studies (*n* = 38).

	Name of social media platforms	Number (percentage)
Data sources	Twitter	21 (55.2%)
	Multisocial media platforms	7 (18.4%)
	Online community	4 (10.5%)
	Facebook	2 (5.3%)
	Reddit	2 (5.3%)
	Sina Weibo	2 (5.3%)

Data size/sample size	< 10,000	11 (28.9%)
	10,000–1,00,000	15 (39.5%)
	> 1,00,000	12 (31.6%)

Language of the analyzed text	English	25 (65.8%)
	Korean	4 (10.5%)
	Chinese	4 (10.5%)
	Turkish	3 (7.9%)
	English + other language	2 (5.3%)

Geographical scope of collected data	USA	5 (13.2%)
	China	4 (10.5%)
	Australia	2 (5.3%)
	Turkey	3 (7.9%)
	Republic of Korea	4 (10.5%)
	Multinational	4 (10.5%)
	Not available	16 (42.1%)

Methods for retrieving SM data	Keywords-based	21 (55.2%)
	Hashtag-based	6 (15.8%)
	User-based	3 (7.9%)
	Column-based	2 (5.3%)
	Combined	4 (10.5%)
	Not available	2 (5.3%)

Inclusion and exclusion criteria	Yes	10 (26.3%)
	No	28 (73.7%)

**Table 3:** Detailed description of NLP in the studies.

Author, year of publication	Study population/study sample	Data collection tool	Data analysis software	Data analysis method	Brief descriptions of the data analysis process	
Al-Garadi et al., 2022	137 million tweets about nonmedical prescription drug use (NMPDU)	NA	NA	Emotion analysis, sentiment analyses, topic modeling Lexicon-based approach VADER: hybrid	(1) Classification model: a previously validated robustly optimized BERT (RoBERTa) was used to classify tweets into NMPDU (potential nonmedical use), consumption, mention, and unrelated. (2) Estimate gender distributions of the NMPDU users using a pre-established NLP text classification model. (3) Emotion analysis: National Research Council (NRC) Sentiment and Emotion Lexicons. (4) Sentiment analyses of NMPDU tweets: using VADER (an open-source Twitter sentiment model) that assigns numerical sentiment scores between −1 and 1 (5) Personal and social concern analysis: using the validated linguistic inquiry and word count (LIWC) to characterize words into psychologically meaningful categories. (6) Statistical testing: the Mann–Whitney *U* test was used to compare outcomes between two independent groups. (7) Topic modeling: the LDA algorithm was used. A qualitative manual examination was performed to group the results	
Al-Garadi et al., 2022	6348 tweets related to intimate partner violence	Python	NA	Using traditional and advanced algorithms for text classification	(1) Manual annotation of the tweets into two categories (2) Preprocessing: manual (3) Three traditional machine learning algorithms (decision tree, SVM, and neural network) were used to construct the classifier; more advanced deep learning–based algorithms for text classification including bidirectional long short–term memory (BiLSTM) and two transformer-based models (BERT and RoBERTa) were used to experiment the model; model training validation (4) Postclassification analyses: learning curve analysis, error analysis, and bias analysis	
Chen et al., 2022	1,69 Reddit posts related to the stigma experience of alcohol, cannabis, and opioid use	pushshift.io API	Python	Topic modeling	(1) Annotation of stigma constructs using directed content analysis; compare stigma types using word clouds (2) Data preprocessing: Python *spaCy* was used (3) Topic modeling: non-negative matrix factorization (NMF) was performed using Python *scikit-learn* package to identify common topics; heatmap visualization was performed to examine the expression of affective and temporal factors in multiple topics and with different substances. (4) Estimation of the prevalence of temporal and affective factors: WordNet-affect lexicon was used. (5) Hierarchical clustering of the data by topics and contextual factors; illustrate the salience of affective and temporal factors through heatmap visualization; Python *Matplotlib* and *Seaborn* packages were employed	
Doğan et al., 2023	78,162 tweets related to anti-LGBTI + hate speech	Python (Tweepy library)	Python	Sentiment analysis and qualitative content analysis	(1) Sentiment analysis: NLP algorithm was used to classify tweets into positive, neutral, or negative (2) Qualitative content analysis of 556 most liked tweets	
Elyashar et al., 2021	16,616,970 tweets related to the state of mind of healthcare professionals from 53,063 Twitter accounts during the COVID-19 pandemic	Twitter API	Python, R	Topic modeling and sentiment analysis hybrid ML approach: neural networks		(1) Preprocessing: Natural Language Toolkit (*NLTK*) library and *WordNet* in Python were used. (2) Topic modeling: the LDA algorithm was used. (3) Topic distribution analysis: quantify topic distribution using the coherent score and *Jaccard* similarity index (using the multiple correspondence analysis algorithm from the *Jaccard* package in the R language). (4) Sentiment analysis: VADER that assigns scores ranging from −1∼1 was used. (5) Emotional analysis: the pretrained RNN model was used to quantify the probabilities of Ekman's six basic emotions. (6) Emotional trends and correlation analysis between the tweet sentiment and new COVID cases: Welch *t*-test, Mann–Whitney U test, and Shapiro–Wilk test
Ford et al., 2022	1723 Reddit posts forwarded by nurses and physicians during the COVID-19 pandemic	*R* (RedditExtractoR package)	Mallet software, R, and SPSS	Topic modeling and inferential statistics	(1) Preprocessing: manual (2) Topic modeling: the LDA model was trained with the dataset; evaluate the performance of the model by computing the perplexity statistic on the validation set. LDA analyses were performed using the MALLET software (3) Semantic coherence of emerging topics: examined visually with word clouds generated using the *word cloud* package in *R.* (4) Examine the prevalence of topics over time: a) examine the overall distribution of emerging topics by computing the mean topic proportion score; b) identify differences in prevalence for nurses versus physicians through multiple linear regression	
Göksel et al., 2023	13,042 tweets related to autism spectrum disorders	Python (Tweepy library)	NA	Emotional analysis and qualitative analysis; ML transformers network	(1) Emotional analysis: BERT variation BERTurk was used to perform feature extraction; classification was performed with multilayer perceptron (MLP) (2) Qualitative analysis: Colaizzi's phenomenological interpretation method was used	
Guo et al., 2021	1148 tweets related to the experience of COVID-19-positive individuals	R (*rtweet* package)	R	Topic modeling	(1) Data preprocessing: redundancy removal, stemming (manual/NLP not clear) (2) Topical themes extraction: The preferred number of latent topics was determined by using the minimization approach; the LDA algorithm was used to generate the representative word list for each topic and the value of the probability distribution of topics for each tweet. (3) Interpret the theme of the latent topic: LDAvis (a visualization data analysis method) was used. (4) R Packages *rtweet, tm, topicmodels, LDAvis,* and *ldatuning* were used	
Guo et al., 2019	56,931 tweets related to public perceptions of nursing	RStudio software	R	Sentiment analysis and topic modeling; lexicon-based approach	(1) Preprocessing: a document-term matrix was generated (2) Sentiment analysis: NRC word-emotion association lexicon (EmoLex) was used to categorize the sentiments and emotion types of tweets; performed using R *tidytext* package (3) Topic modeling: the harmonic mean method (a Bayesian probabilities approach) was used to estimate the optimal number of topics; LDA algorithms were used to group tweets based on co-occurring words into topics. (4) Name each topic through manual examination. (5) Present the results of topic modeling using *LDAvis* R package	
Jiang et al., 2023	14,060 online posts about nurse' discussions related to workplace stressors	Java (HtmlUnit package)	Python	Topic modeling and thematic qualitative analysis	(1) Preprocessing: Python *NLTK* library was used. (2) Topic modeling: the LDA algorithm was used to identify the relationships among the text; *MALLET* package for Python was used to perform statistical NLP, clustering, and topic modeling; Python *Gensim* library was used to build the topic models. (3) Thematic qualitative content analysis: an interpretive social science approach	
Kong et al., 2022	9,83,039 Sina Weibo posts related to the public discourse and sentiment toward dementia	NA	Python	Topic modeling, semantic network analyses, and sentiment analysis; lexicon-based approach	(1) Text preprocessing: word segmentation was performed using *Jieba* Python toolkit; tokenization was performed using Python packages *Pickle, CountVectorizer,* and *TfidfVectorizer*. (2) Topic modeling: the LDA algorithm was used to identify dementia-related themes; key topics were chosen based on the results of LDA modeling; manual examination and coding topics. (3) Semantic network analysis was performed to cross-validate the results from topic modeling and characterize public perceptions of dementia using Gephi. (4) Sentiment analysis: two emotion dictionaries (the Chinese DLUT-emotion ontology and the Chinese emotion valence dictionary) in conjunction with dictionary-based techniques were used	
Koren et al., 2021	1,10,993 Facebook posts and comments related to the public sentiment of nurses during COVID-19	Python	Python	Sentiment analysis and information retrieval; ML transformers network	(1) Preprocessing: background noise was removed (manual/NLP not clear) (2) Topic modeling and sentiment analysis: BERT algorithm was performed through the Python library *Hugging Face* to detect sentiments, along with the frequency. (3) Using information retrieval method to label posts: Python-based information retrieval tool Whoosh (*Anserini*) was used to detect the additional topics	
Kwok et al., 2021	31,100 tweets related to COVID-19 vaccination	R	R	Topic modeling and sentiment analysis; lexicon-based approach	(1) Preprocessing: R library packages of *qdapRegex and tm*; noise removal and stemming (2) Word token association calculation: R library package *widyr* (3) Topic modeling: the LDA algorithm (performed through R library package *ldatuning*) was used to estimate the optimal number of topics; word cloud was used to visualize the top 100 words. (4) Sentiment analysis: scoring each tweet based on NRC word-emotion association lexicon using R library package *Syuzhet*	
Lee et al., 2022	12,413 tweets related to dementia/Alzheimer's	NCapture software	Python	Qualitative content analysis, and topic modeling	(1) Qualitative content analysis: manual coding; themes extraction (2) Data preprocessing: stop word removal (3) Topic modeling: the LDA algorithm was used to extract themes. (4) Comparison: compare the extracted themes derived from manual coding and topic modeling	
Lee et al., 2020	7,54,744 SM posts related to information needs and emotions related to cancer	Crawler	NA	Natural language processing	(1) Development and evaluation of a cancer ontology: determine domain and scope of the ontology; identify existing ontologies on cancer and an emotion ontology; terms extraction; using NLP to extract consumer terms related to cancer; define the classes and their hierarchy and relationships; evaluate the structure, correctness, and quality of the ontology. (2) Analysis of social media data using the ontology: ontology-based NLP was performed to extract class concept; frequency of specific cancer was counted	
Lee et al., 2019	4,40,000 tweets related to health technology	Python	Python and R	Sentiment analysis; lexicon-based approach	(1) Keywords frequency analysis using R. (2) Sentiment analysis: sentiment dictionary SentiWordNet was used to classify the sentiment into positive or negative. (3) Sentiment scores were calculated	
Lee et al., 2022	50,626 SM posts related to the symptom experience of ovarian cancer patients	NA	R	Topic modeling	(1) Data preprocessing: redundancy removal, stop word removal, and stemming (2) Manual selection of the top five most frequently discussed symptoms and their relating posts. (3) LDA: the LDA model was conducted using the selected posts. (4) Manual examination was performed to decide the final topic names	
Lee et al., 2022	853 SM posts related to the needs of ovarian cancer patients and caregivers	NA	Python	Machine learning classifies model development	(1) Needs annotation: 12 types of needs were generated according to literature; each post was annotated accordingly; the frequency of each need type was calculated. (2) Machine learning model that predict the specific need type was developed using bag-of-words (BOW) presentation. (3) Performance of the classification model was evaluated using macro F1 score	
Luo et al., 2023	5112 Weibo posts related to the early experiences of frontline nurses at the beginning of the COVID-19 pandemic	Python	Python and NVivo	Text mining, qualitative analysis, descriptive statistics, and sentiment analysis	(1) Text preprocessing: *Jieba* Python toolkit was used for word segmentations. (2) Text mining and sentiment analysis: Bi-LSTM network deep learning model was used to extract user sentiment features; a six-category sentiment analysis deep learning model was constructed based on a pretrained RoBERTa-BiLSTM architecture. (3) Model evaluation: macro F1 and accuracy (ACC) rate were used to evaluate the effects of the model. (4) Qualitative analysis was conducted using NVivo	
Mermer et al., 2022	10,308 tweets related to COVID-19 vaccination	Python (twint library)	Python	Sentiment analysis; lexicon-based approach and ML approach	(1) Data preprocessing: Python *NLTK* library was used. (2) Sentiment analysis: dictionary-based methods (*Pandas, NumPy, Matplotlib, NLTK, Scikit-learn, and TextBlob* libraries in Python) were used to classify a tweet's hashtag as positive, negative, or neutral; ML classifier was trained and tested with Naive Bayes (NB), random forest (RF), XGBoost (XGB), and logistic regression (LR) algorithms. (3) Model performance evaluation: count vectors accuracy, word-level TF-IDF accuracy, N-Gram TF-IDF accuracy, and char-level accuracy were used to evaluate the accuracy values	
Min et al., 2023	9155 diabetes self-care-related questions posted on an online diabetes-related community	NA	Python	Topic modeling	(1) Data preprocessing: pruning, tokenization, normalization, and stop word removal using Python packages such as *PyKoSpacin and, MeCab* (2) Document embedding: using a type of KoBERT, i.e., Ko-sentence-transformers model (3) Document clustering: the uniform manifold approximation and projection (UMAP) algorithm was used to reduce the dimensionality of local structure; hierarchical density–based spatial clustering of applications with noise (HDBSCAN) was used for clustering. (4) Qualitative analysis: Interpret topics derived from topic modeling	
Miller et al., 2022	67,433 tweets related to nursing profession online presentation and nurses' experience during COVID	NA	NA	Topic modeling	(1) Word adjacent modeling (2) Text preprocessing: tokenization and stop word removal (3) Graph modularity algorithm: Louvain algorithm (4) Manual content analysis	
Odlum et al., 2020	1763 tweets related to COVID-19	Python	ORA	Topic modeling, sentiment analysis, and qualitative content analysis	(1) COVID-19 related tweets were randomly extracted. (2) NLP and a clustering Newman algorithm were applied to the publicly available tweet corpus. (3) A network diagram was used to visualize 15 topics. (4) Qualitative thematic analysis was performed on the detected topics	
Odlum et al., 2018	68,736 tweets related to the health information needs about Ebola	NCapture software	Notepad++ and Weka	Natural language processing	(1) Data cleaning: dimensionality was reduced and text was transformed to a vector form and N-gram through cleaning, stemming, and redundancy removal using Weka. (2) A tweet term-frequency dictionary was computed with the N-gram method. (3) Topics were detected and summarized through descriptive statistics, visualization (using infographics), classification, and clustering (K-means algorithm)	
Park et al., 2020	321,339 SM posts related to the public's emotions about cancer and factors affecting emotions	SK telecom Smart insight	R	Association rule mining and social network analysis	(1) Data preprocessing (2) Code the posts based on four emotional groups (3) Frequency analysis (4) Association rule mining: applying the Apriori algorithm to investigate the relationship between different emotion-related factors and emotion groups. (5) Social network analysis: examine and visualize relationships between emotion groups or emotion-related factors that appeared together in each post in the two groups	
Patel et al., 2022	269,747 tweets related to advance care planning and specific life-sustaining interventions	NA	MATLAB and SPSS	Topic modeling and sentiment analysis	(1) Text preprocessing: text parsing (2) Topic modeling: LDA (3) Sentiment analysis: VADER and lexicon-based approach	
Shin et al., 2021	12,302 SM posts related to public opinions and perceptions about nursing	Collection engine such as crawlers	Sometrend	Text mining and opinion mining; lexicon-based approach	(1) Data cleansing: not clear (2) Text mining: performed through NLP techniques such as morpheme analysis, part-of-speech tagging and postprocessing, syntax chunking, syntax analysis, and semantic analysis; frequency of keywords was analyzed. (3) Opinion mining: sentiment dictionary was used to classify emotional words. (4) Social network analysis: related word analysis was conducted to identify top 25 high frequency words; related word analysis was achieved through syntax analysis based on core NLP. (5) Results were visualized through Tag cloud and Word cloud	
Smith et al., 2021	6317 tweets related to heat illness	Postman software	R	Sentiment analysis and topic modeling; lexicon-based approach	(1) Data preprocessing: lemmatization using R package *tm* (2) Term frequency analysis: *tidytext* and *word cloud* packages in R were used. (3) Sentiment analysis: NRC word-emotion association lexicon was used to identify the sentiments and emotions; *Syuzhet* package in R was used. (4) Topic modeling: the LDA algorithm was used through R *LDAvis, stringi, rjson, tm,* and *topicmodels;* topic optimization methods were used through *psych* package and the harmonic means method, and elbow plots were produced using the *ldatuning* package.	
Song et al., 2022	1733 blog posts related to adolescents' diet behaviors	Python (Selenium and pandas module)	Python	Text mining and semantic network analysis	(1) Data extraction and preprocessing: word extraction; TF-IDF calculation; selection of top 50 words; document-term matrix generation; binary co-occurrence matrix generation; *KoNLPy* and *pandas* module in Python were used. (2) Semantic network analysis was performed to understand the relationship between refined words related to adolescents' diet. *KoNLP* and *pandas* module was used to conduct frequency analysis, centrality analysis, and CONCOR analysis. (3) Mutually exclusive subgroups in the semantic network were identified using convergence analysis	
Tokac et al., 2022	582,399 tweets related to nursing during COVID-19	NA	R	Sentiment analysis and natural language processing; lexicon-based approach	(1) Data preprocessing: redundancy removal (2) Sentiment analysis: NRC word-emotion association lexicon was used to identify the opinions and emotions (3) Network graph was then used to display the relationship between unique words of the tweets. (4) R Packages *academictwitteR, tidytext, and ggplot2* were used	
Wu et al., 2023	1000 SM posts related to suicide	NA	NA	Classification model development and evaluation	(1) Manual annotation: annotate the suicide-related SM posts as low, medium-, or high-risk. (2) Text classification model construction and validation: using simple RNN and BERT to conduct text classification; validate the BERT model by comparing BERT results against the two manual annotation	
Xavier et al., 2022	13,868 tweets related to the sentiments and emotions of nurses during COVID-19	A pre-established database was used.	R and JMP Pro	Sentiment analysis; lexicon-based approach	(1) Data cleaning: stop word removal (2) Sentiment analysis: sentiment scores were calculated using AFINN, Bing, and NRC word-emotion association lexicon (3) A word cloud of sentiments was presented	
Xue et al., 2022	34,885 Facebook posts and 51,835 comments related to heart disease and heart health	*Selenium* Python, CrowdTangle, and Facepager	LIWC software and Python	Topic modeling and sentiment analysis; lexicon-based approach	(1) Sentiment analysis: linguistic inquiry and word count (LIWC) was used to obtain the sentiments of the posts and comments. (2) Topic modeling: the LDA algorithm was used through *Gensim* in Python (3) Statistical analysis: analyze and compare the level of emotions in posts and comments using 2-tailed 2-sample *t* tests	
Yang et al., 2023	152,670 tweets related to self-reported chronic stress experiences	Twitter streaming API	Python	Classification model development and evaluation	(1) Manual annotations: manually annotated the tweets as positive or negative (2) Classification model construction and experiment: 5 traditional classification algorithms (Gaussian, Naïve Bayes, SNM, random forest, KNN, and shallow neural network) and 2 advanced classification algorithms (RNN with BiLSTM and BERT). (3) Postclassification analyses: classification errors identification; learning curve analysis	
Yoon et al., 2022	58,094 tweets related to Alzheimer's disease and related dementia caregiving topics	NCapture, ORA software, and Python	Pearl	Topic modeling and sentiment analysis; lexicon-based approach	(1) Newman clustering algorithm was used to group associated tweets in the corpus. (2) Compare emotional valence scores of tweets from before and after the COVID-19 pandemic. (3) Manual examination was performed to detect topics	
Yoon et al., 2022	12,413 tweets related to dementia/Alzheimer's	NCapture	R	Manual sentiment analysis and machine learning–based sentiment analysis; lexicon-based approach	(1) Manual sentiment analysis was conducted by emotional valence score assignment. (2) NLP-based sentiment analysis: Text preprocessing; R packages (*Afinn, Syuzhet, and Bing*) were used to extract an emotional valence score for each tweets. (3) One-way ANOVA was used to compare the aggregated mean emotional valence scores from manual coding and the mean scores produced by machine learning sentiment analysis.	
Zhou et al., 2023	10,186 tweets related to emotional responses during the COVID-19 pandemic forwarded by nurses and nursing students	Python	NA	Topic modeling and emotional analysis; ML approach	(1) Preprocessing: not specific (2) Emotion analysis: a pre-established deep learning algorithm that was trained with a RNN was used to predict six basic emotions (anger, disgust, fear, joy, sadness, and surprise) from tweets. (3) Topic modeling: the LDA algorithm was used to identify the topics that were associated with nurses' and nurse students' emotions.	
Zhou et al., 2024	2823 social media posts related to nurse prescribing	Python	NA	Topic modeling and sentiment analysis	(1) Personnel analysis: identify the professional categories of users; (2) Topic analysis: Bayesian LDA models was used to explore public and medical professionals' concerns. (3) Sentiment analysis: SnowNLP, Bayesian LDA model, and BosonNLP (based on dictionary-based approach) were used to explore public and medical professionals' attitudes. (4) Qualitative content analysis	

*Note:* National Research Council Sentiment and Emotion Lexicons (NRC Sentiment and Emotion Lexicons) is a collection of seven lexicons, including the widely used NRC word-emotion association lexicon. **NRC** word-emotion association lexicon is a list of English words and their associations with eight basic emotions (anger, fear, anticipation, trust, surprise, sadness, joy, and disgust) and two sentiments (negative and positive). MALLET: a Java-based package for statistical natural language processing, document classification, clustering, topic modeling, information extraction, and other machine learning applications to text.

Abbreviations: BERT, bidirectional encoder representations from transformers; BiLSTM, bidirectional long short–term memory; KNN, K-nearest neighbor; LDA, latent Dirichlet allocation; LIWC, linguistic inquiry and word count; NA, not available; NLTK, Natural Language Toolkit; RNN, recurrent neural network; RoBERTa: robustly optimized bidirectional encoder representations from transformers; SVM, support vector machine; VADER, valence aware dictionary and sentiment reasoner.

## Data Availability

The datasets used in the current study are available from the corresponding author on reasonable request.
